# Suprainguinal Fascia Iliaca Block as Primary Anesthesia in a Child-Pugh C Patient Undergoing Percutaneous Hip Fixation: A Case Report

**DOI:** 10.7759/cureus.100018

**Published:** 2025-12-24

**Authors:** Joaquim Borba, Francisco Flor de Lima Machado, Ricardo Lima, Paulo Frias, Rui Silva, João Borges

**Affiliations:** 1 Anesthesiology, Hospital do Divino Espírito Santo, Ponta Delgada, PRT

**Keywords:** decompensated liver cirrhosis, hip fracture, regional anesthesia, remifentanil, suprainguinal fascia iliaca block

## Abstract

Hip fracture surgery in patients with decompensated liver cirrhosis is associated with substantial perioperative morbidity and mortality. The anesthetic management of these patients is complicated by coagulopathy, thrombocytopenia, and a hyperdynamic circulatory state. These pathophysiological changes often contraindicate neuraxial techniques due to the risk of spinal hematoma, while general anesthesia poses significant risks of intraoperative hypotension and delayed recovery due to impaired hepatic metabolism. We present the case of a 57-year-old female with alcohol-related Child-Pugh C cirrhosis who presented with an unstable pertrochanteric femoral fracture. Despite attempts at optimization, preoperative laboratory evaluation demonstrated severe anemia, thrombocytopenia, and coagulopathy (hemoglobin 7.1 g/dL, platelet count 50×10⁹/L, international normalized ratio (INR) 1.7; model for end-stage liver disease-sodium (MELD-Na) score 23), precluding neuraxial techniques. Given her clinical status, a decision was made to utilize a suprainguinal fascia iliaca nerve block (SFINB) as the primary anesthetic technique, supplemented with a remifentanil infusion for analgosedation. This approach allowed for surgical fixation of the fracture while maintaining hemodynamic stability (mean arterial pressure 106-125 mmHg throughout the procedure) without the need for vasopressors. Remifentanil was selected for its organ-independent metabolism, minimizing the risk of drug accumulation. The patient remained awake and cooperative, and the early postoperative course was free of complications. This case illustrates that SFINB, combined with remifentanil-based analgosedation, offers a feasible and safe alternative to general or neuraxial anesthesia in carefully selected high-risk patients with advanced liver disease.

## Introduction

Hip fracture surgery is associated with substantial morbidity and mortality, particularly in frail or comorbid patients with advanced systemic disease [1]. Advanced liver cirrhosis, in particular, poses significant anesthetic challenges. The liver plays key roles in drug metabolism, synthesis of plasma proteins and coagulation factors, regulation of glucose homeostasis, and detoxification of ammonia. In cirrhosis, impairment of these functions, combined with systemic vasodilation and a resulting hyperdynamic circulation, leads to coagulopathy, hypoalbuminemia, and hemodynamic instability [2]. These alterations profoundly affect anesthetic pharmacokinetics and cardiovascular responses, increasing perioperative risk even during minor procedures [2].

As such, anesthetic management of patients with decompensated cirrhosis is particularly complex. Although cirrhosis is now understood as a state in which reductions in procoagulant factors are counterbalanced by concomitant decreases in anticoagulant pathways, such as protein C, protein S, and antithrombin, this state of “rebalanced” hemostasis results in a fragile equilibrium between the risks of both bleeding and thrombosis [3]. Nonetheless, clinically significant coagulopathy and thrombocytopenia remain highly relevant in procedural decision-making, and neuraxial techniques are often avoided due to the potentially catastrophic consequences of neuraxial hematoma in the setting of impaired clot formation and limited physiological reserve [4]. Conversely, general anesthesia (GA) may exacerbate hypotension and impair hepatic perfusion due to systemic vasodilation and myocardial depression. Additionally, reduced hepatic metabolism and altered protein binding can prolong anesthetic effects and increase sensitivity to sedatives and opioids, risking delayed recovery and encephalopathy [2].

Regional anesthesia techniques targeting the peripheral nerves of the lower limb have emerged as valuable alternatives in such high-risk patients with lower limb fractures, including femoral neck fractures. The suprainguinal fascia iliaca nerve block (SFINB) provides broad coverage of the femoral, lateral femoral cutaneous [5], and aims to target the obturator nerves by facilitating cephalad spread of local anesthetic [6]. Nevertheless, while SFINB increases the likelihood of obturator nerve coverage in comparison to the standard approach, complete blockade remains variable and cannot be guaranteed in all patients, requiring adequate analgosedation and back-up planning. By achieving blockade of these nerves, the technique provides effective anesthesia for the hip joint, the femoral fracture site, and the site of incision. This enables anterior hip procedures, such as percutaneous fixation [7], to be performed while minimizing systemic hemodynamic disturbances and avoiding the coagulation-related risks associated with neuraxial techniques.

We present the case of a patient with Child-Pugh C cirrhosis and significant coagulopathy undergoing percutaneous fixation of an unstable pertrochanteric fracture. In this scenario, we employed the SFINB as the primary anesthetic technique, supplemented only by remifentanil-based analgosedation. This approach distinguishes our case from standard practice, where SFINB is typically utilized merely as an analgesic adjunct to general or neuraxial anesthesia. This successful application highlights the feasibility of SFINB as a definitive anesthetic strategy when traditional options pose prohibitive risks. This article was previously presented as a poster at “Euroanaesthesia 2025” on May 27, 2025, in Lisbon.

## Case presentation

A 57-year-old female with a history of ethanol-related liver cirrhosis, classified as Child-Pugh C ( model for end-stage liver disease-sodium, MELD-Na, score 23), presented to the emergency department following a fall that resulted in an unstable pertrochanteric fracture of the right femur. Her medical history included chronic anemia, splenomegaly, ascites, peptic ulcer disease (*Helicobacter pylori*-negative), as well as esophageal varices, requiring a recent hospitalization in the Gastroenterology unit due to upper gastrointestinal tract bleeding.

On admission, the patient was hemodynamically stable and exhibited no signs of hepatic encephalopathy. Laboratory studies revealed severe anemia, thrombocytopenia, and coagulopathy (Table 1). The patient weighed around 80 kg and her American Society of Anesthesiologists (ASA) physical status was classified as IV. Following admission (hemoglobin, Hb, 6.4 g/dL), her clinical course was complicated by an acute decline in Hb to a nadir of 4.1 g/dL. This necessitated urgent transfusion of red blood cells, as well as fresh frozen plasma and albumin. By the preoperative assessment, these measures had successfully raised her Hb to 7.1 g/dL and improved the international normalized ratio (INR) to 1.7, though significant thrombocytopenia persisted (50 × 10⁹/L), prompting immediate preoperative platelet transfusion. Despite optimization, her Child-Pugh score remained unchanged compared to admission, and the recalculated MELD-Na score was 22 (from 23 on admission). Additionally, a right subclavian central venous catheter was placed to facilitate hemodynamic monitoring and blood product administration.

**Table 1:** Admission and preoperative laboratory values INR: International normalized ratio.

Parameter	Reference range	On admission	Preoperative
Hemoglobin (g/dL)	12–16	6.4	7.1
Platelet count (×10⁹/L)	150–400	70	50
INR	0.9–1.2	2.1	1.7
Albumin (g/dL)	3.5–5.0	1.5	1.8
Total bilirubin (mg/dL)	<1.2	4.35	4.55
Creatinine (mg/dL)	0.6–1.2	0.7	0.9
Sodium (mmol/L)	135–145	134	132

In the operating room, ASA standard monitoring was applied, and an invasive arterial line was placed for continuous blood pressure monitoring and blood sampling. Due to the clear contraindications to neuraxial anesthesia and the hemodynamic risks associated with general anesthesia, the decision was made to perform a suprainguinal fascia iliaca nerve block (SFINB) as the primary anesthetic technique.

With the patient in the supine position, a high-frequency linear ultrasound probe was placed in a sagittal orientation medial to the anterior superior iliac spine (ASIS) to identify the iliacus muscle, the fascia iliaca, and the internal oblique muscle. Using an in-plane approach from caudal to cranial, an 80 mm echogenic needle was advanced until the tip penetrated the fascia iliaca. Hydrodissection confirmed the correct plane between the fascia iliaca and the iliacus muscle. A total of 40 mL of 0.5% ropivacaine was administered, and real-time ultrasound visualization confirmed cephalad spread of the local anesthetic within the iliac fossa. Sensory blockade was assessed 15 minutes after injection using cold discrimination. Loss of sensation was confirmed in the femoral nerve territory (anterior thigh), lateral femoral cutaneous nerve territory (lateral thigh), and medial thigh, consistent with obturator nerve involvement.

Analgosedation was provided by a remifentanil infusion, titrated to effect with a maximum dose of 0.05 µg/kg/min. Supplemental oxygen (2 L/min) was delivered via a nasal cannula with continuous capnography to ensure continuous monitoring of ventilation. The patient remained awake, responsive, and cooperative, maintaining a Richmond Agitation-Sedation Scale (RASS) score of 0 to -1 throughout the procedure. Airway management equipment and induction agents were immediately available to address potential respiratory depression or the need for conversion to general anesthesia. Surgical fixation was performed using a Gamma3 trochanteric nail without complications. Estimated blood loss was approximately 800 mL. This was managed intraoperatively with two units of red blood cells, two units of fresh frozen plasma, and one pool of platelets. In response to this active blood loss, 1 g of tranexamic acid was administered to target the hyperfibrinolytic component of cirrhotic coagulopathy. Hemodynamic stability was maintained throughout the two-hour procedure without the need for vasopressors; mean arterial pressure (MAP) ranged between 106 and 125 mmHg, maximum lactate was 1.44 mmol/L, and urine output was maintained at > 1 mL/kg/h, indicating adequate end-organ perfusion.

Following surgery, the patient was transferred to a high-dependency unit for close monitoring. Her postoperative course was notable for stable hemodynamics and the absence of local anesthetic toxicity or neurological complications. After two days in the high-dependency unit, the patient was discharged to the orthopedic ward, where care was continued.

## Discussion

Cirrhosis is associated with severe coagulopathy through multiple mechanisms [2,3]. Not only is the hepatic synthesis of clotting factors (Factors II, V, VII, IX, X, and XI) markedly reduced, but thrombocytopenia and platelet dysfunction are also common due to hypersplenism, decreased thrombopoietin production, and impaired platelet aggregation, as well as dysregulated fibrinolysis [3]. In advanced cirrhosis, reduced intestinal absorption of vitamin K, often due to cholestasis, decreased bile salt secretion, or poor nutritional status, can further impair the synthesis of vitamin K-dependent clotting factors, worsening coagulopathy [8].

Given this degree of coagulopathy and thrombocytopenia, neuraxial anesthesia would have carried an unacceptably high risk of neuraxial hematoma. The patient’s platelet count of 50x10^9^ and elevated INR of 1.7 were below commonly recommended thresholds for safe spinal or epidural anesthesia [2,4]. In the setting of advanced cirrhosis, impaired clot formation and fragile hemostasis further increase the potential for catastrophic bleeding in the confined neuraxial space, making neuraxial techniques unsuitable for this case.

Cirrhosis is also characterized by a hyperdynamic circulatory state, marked by systemic vasodilation, low systemic vascular resistance, and increased cardiac output. These patients have limited ability to tolerate fluctuations in preload or afterload and are highly susceptible to hypotension during anesthesia. In addition, altered hepatic metabolism and reduced protein binding can potentiate the effects of anesthetic agents. Hypoalbuminemia increases the biologically active free fraction of highly protein-bound drugs [9], while reduced cytochrome P450 activity significantly impairs the clearance of many opioids and sedatives. Together, these disturbances markedly narrow the therapeutic window of general anesthesia in advanced liver disease.

With these challenges in mind, we opted for performing the suprainguinal fascia iliaca nerve block (SFINB). This technique involves depositing local anesthetic above the inguinal ligament, deep to the fascia iliaca. This approach promotes cephalad spread of the injectate, ensuring coverage of the femoral and lateral femoral cutaneous nerves while facilitating spread to the obturator nerve.

Clinically, this offers two main advantages for the high-risk cirrhotic patient. First, the block is performed in a superficial, compressible region, mitigating the hemorrhagic risks inherent to neuraxial or deep blocks. Second, SFINB spares the sympathetic chain. Unlike spinal or epidural anesthesia, which induces neuraxial sympathectomy, this technique avoids the consequent systemic vasodilation and hypotension. This preservation of vascular tone is critical in cirrhotic patients, who often manifest a hyperdynamic circulation and limited cardiovascular reserve.

Finally, the potential for local anesthetic systemic toxicity (LAST) was a relevant concern given the patient’s marked hypoalbuminemia (1.8 g/dL), which increases the biologically active unbound fraction of ropivacaine [9]. Although a relatively high volume (200 mg) was administered, this dose corresponded to approximately 2.5 mg/kg for this 80 kg patient, remaining well within established safety limits [10]. To further mitigate risk, the block was performed under ultrasound guidance using slow incremental injection with frequent aspiration to prevent intravascular entry. Furthermore, 20% lipid emulsion was immediately available for rescue therapy. Ultimately, no signs of systemic toxicity occurred intraoperatively or postoperatively, supporting the safety of this technique in cirrhotic patients when appropriate precautions are taken.

Furthermore, the choice of sedative agent was critical to addressing the pharmacokinetic challenges. To mitigate the risks of reduced hepatic clearance, remifentanil was selected for analgosedation. Unlike other opioids or sedatives that rely on hepatic metabolism, remifentanil is hydrolyzed by non-specific plasma esterases [11]. This organ-independent metabolism prevents drug accumulation and ensures rapid recovery, minimizing the risk of postoperative respiratory depression or the precipitation of hepatic encephalopathy.

## Conclusions

This case report highlights the suprainguinal fascia iliaca nerve block (SFINB), as a feasible primary anesthetic alternative to neuraxial or general anesthesia in patients with advanced liver cirrhosis. By successfully addressing the critical challenges of coagulopathy, altered drug metabolism, and hemodynamic vulnerability, this approach enabled safe surgical fixation without the need for vasopressor support or perioperative complications. This strategy offers a valuable alternative for high-risk patients in whom standard anesthetic techniques pose prohibitive risks.
